# A novel preoperative inflammation score system established for postoperative prognosis predicting of intrahepatic cholangiocarcinoma

**DOI:** 10.1186/s12885-023-10668-x

**Published:** 2023-02-24

**Authors:** Jun Fu, Qinjunjie Chen, Zisen Lai, Kongying Lin, Guoxu Fang, Zongren Ding, Yuzhen Gao, Yongyi Zeng

**Affiliations:** 1 Department of Hepatopancreatobiliary Surgery, Mengchao Hepatobiliary Hospital of Fujian Medical University, 312 Xihong Road, Fuzhou, China; 2 Department of Hepatic Surgery IV, the Eastern Hepatobiliary Surgery Hospital, Naval Medical University, Shanghai, China; 3 Department of Clinical Laboratory, Sir Run Run Shaw Hospital, Zhejiang University School of Medicine, Hangzhou, China

**Keywords:** Systemic inflammation, Intrahepatic cholangiocarcinoma, Liver resection, Prognosis

## Abstract

**Background:**

Inflammation is implicated in tumorigenesis and has been reported as an important prognostic factor in cancers. In this study, we aimed to develop and validate a novel inflammation score (IFS) system based on 12 inflammatory markers and explore its impact on intrahepatic cholangiocarcinoma (ICC) survival after hepatectomy.

**Methods:**

Clinical data of 446 ICC patients undergoing surgical treatment were collected from the Primary Liver Cancer Big Data, and then served as a training cohort to establish the IFS. Furthermore, an internal validation cohort including 175 patients was used as internal validation cohort of the IFS. A survival tree analysis was used to divide ICC patients into three groups (low-, median-, and high- IFS-score groups) according to different IFS values. Kaplan-Meier (KM) curves were used to compare the overall survival (OS) and recurrence-free survival (RFS) rates among three different groups. Cox regression analyses were applied to explore the independent risk factors influencing OS and RFS.

**Results:**

In the training cohort, 149 patients were in the low-IFS-score group, 187 in the median-IFS-score group, and 110 in the high-IFS-score group. KM curves showed that the high-IFS-score group had worse OS and RFS rates than those of the low- and median-IFS-score groups (P < 0.001) in both the training and validation cohorts. Moreover, multivariable Cox analyses identified high IFS as an independent risk factor for OS and RFS in the training cohort. The area under the curve values for OS prediction of IFS were 0.703 and 0.664 in the training and validation cohorts, respectively, which were higher than those of the American Joint Committee on Cancer (AJCC) 7th edition TNM stage, AJCC 8th edition TNM stage, and the Child-Pugh score.

**Conclusion:**

Our results revealed the IFS was an independent risk factor for OS and RFS in patients with ICC after hepatectomy and could serve as an effective prognostic prediction system in daily clinical practice.

## Introduction

Intrahepatic cholangiocarcinoma (ICC) is one of the deadliest malignancies worldwide, which arises from the epithelial cells of intrahepatic bile ducts [1]. ICC has become the second most common primary liver malignancy after hepatocellular carcinoma (HCC) [2]. In recent decades, the incidence of ICC in the United States has been increasing [1, 3]. Although surgical resection is recommended as the first-line treatment for ICC, unfortunately, only 30–40% of patients had surgical opportunities because most patients have been found to present regional or distant metastases at the time of diagnosis [4].

More importantly, even after curative-intent resection, the long-term survival of ICC is still dismal because of the high postoperative recurrence rate [4]. Selection of appropriate patients for surgery is one method to improve survival prognosis. Preoperative evaluation of the resectability of patients with ICC is usually based on BCLC stage, TNM stage, and Child-Pugh grade. Common prognostic indicators, like tumor size, tumor number, vascular invasion, and lymph node metastasis, are often acquired by accurate postoperative pathological data. Consequently, obtaining parameters through preoperative low-cost hematological examination is significant and cost-effective, which can also be used for survival prognosis prediction. Thus, finding an accurate prognostic biomarker is necessary and urgent for surgeons to evaluate prognosis and guide the choice making of treatment before hepatectomy, which provides ICC patients with reasonable individual treatment.

In 1863, Virchow first reported the relationship between inflammation and tumorigenesis [5]. Inflammation has been found to be involved in the onset and progression of diverse diseases. The use of preoperative inflammatory markers to provide individualized survival estimates has been the focus on many reports [6–11]. It has been confirmed that inflammatory markers played an important role in the progression and prognosis of liver cancer [12, 13]. A series of studies have demonstrated that the systemic inflammation score (SIS), the neutrophil-to-lymphocyte ratio (NLR), and the lymphocyte to monocyte ratio (LMR) were all significantly associated with long-term survival in patients with ICC [14]. Nevertheless, there are no studies that combine several inflammation-based indicators to create an integrated prognostic score system for survival prediction in patients with ICC.

In this study, we aimed to develop a novel inflammatory score system that achieves a good survival prediction performance in patients with ICC after surgery using 12 preoperational inflammatory markers. To this end, we used an internal cohort to validate the survival prediction performance of our inflammatory score system at the same time.

## Methods

### Patients

From January 2010 to December 2013, clinical data of 446 patients with ICC who underwent surgical treatment were collected from the Primary Liver Cancer Big Data in Fujian province [15] and used to form the training cohort. For the internal validation cohort, we collected clinical data of 175 patients with ICC from January 2014 to August 2015 from the same institution. These data were retrospectively reviewed. This study was approved by the Institutional Ethics Committee of the Mengchao Hepatobiliary Hospital. Informed consent was obtained from all patients before surgery in this study.

### Inclusion and exclusion criteria

The inclusion criteria were as follows: (1) good preoperative performance with an ECOG score of 0–2, (2) no records of ICC treatment before surgery, (3) radical resection (R0 resection), (4) no other malignant diseases, (5) Child–Pugh graded A or B7, (6) no evidence of main portal vein, hepatic artery, biliary duct, or inferior vena cava invasion. The exclusion criteria were as follows: (1) incomplete clinicopathological data and (2) loss of follow-up within 30 days.

### Liver resection and definitions

All patients underwent routine preoperative laboratory examinations (routine blood tests, platelets, neutrophils, lymphocyte cell count, tumor serum markers, such as carbohydrate antigen 19–9 [CA19–9], carcinoembryonic antigen [CEA], and serum alpha-fetoprotein [AFP]) and imaging examinations (abdominal ultrasound, contrast-enhanced computerized tomography [CT] scan, and/or magnetic resonance imaging [MRI] of the abdomen). The intent of surgery was to completely remove the macroscopic tumors with adequate resection margins. The detailed surgical procedures are described in our previous studies [16].

The diagnosis of ICC through histopathological examination of surgical specimens was based on the WHO criteria [17]. Major hepatectomy was defined as the removal of ≥ 3 Chouinard’s liver segments. Anatomic resection was defined as liver resections based on systematic removal of Couinaud segment(s) containing the tumor together with the tumor-bearing portal vein and corresponding hepatic territory [18]. R0 resection was defined as the absence of macroscopic and microscopic disease at the surgical margin. Microvascular invasion (MVI) was defined as intraparenchymal vascular involvement identified on histological examination [19].

### Inflammatory markers

By reviewing previous studies, we selected 15 inflammatory markers for our research. They are listed as follows: NLR (neutrophil-to-lymphocyte ratio), PLR (platelet-to-lymphocyte ratio), LMR (lymphocyte-to-monocyte), GPR (gamma‑glutamyl transpeptidase [γ-GT]-to-platelet ratio), SII (systemic immune-inflammation index, defined as platelet times neutrophil/lymphocyte), PNI (prognostic nutritional index), APRI (aspartate aminotransferase-to-platelet ratio index), ANRI (aspartate aminotransferase-to-neutrophil ratio index), ALRI (aspartate aminotransferase-to-lymphocyte ratio index), NγLR ([neutrophil times γ-GT]/lymphocyte), dNLR (derived neutrophil-to-lymphocyte ratio, defined as neutrophil/[white cell count - neutrophil count]), PMLR (platelet‑monocyte‑lymphocyte ratio), NMLR (neutrophil‑monocyte‑lymphocyte ratio), SIS (inflammation score, defined as combination of preoperative serum CA19-9 and LMR. Grade I, CA19-9^Low^/LMR^High^; grade II, CA19-9 ^High^/LMR^High^ and CA19-9 ^Low^/LMR ^Low^; grade III, CA19-9^High^/LMR ^Low^) [20], and GAR (globulin-to-albumin ratio).

### Follow-up

All patients were followed up regularly after discharge using the protocol described previously [21]. The relapse of ICC was diagnosed using the same criteria as the initial disease diagnosis, and a multidisciplinary approach was used to deal with the recurrence lesions [22]. Overall survival (OS) and recurrence-free survival (RFS) were defined as endpoints. OS was defined as the interval between the date of LR to the date censored, the date of the patient’s death, or last follow-up. RFS was measured from the date of LR to the date of the first ICC recurrence or last follow-up. This study censored the follow-up on August 30, 2020.

### Cut-off value and inflammation score (IFS)

In this study, we stratified NLR, PLR, LMR, GPR, SII, PNI, APRI, ANRI, ALRI, NγLR, dNLR, PMLR, NMLR, SIS, and GAR into two groups based on receiver operating characteristic (ROC) curves. The optimal threshold values of the highest Youden index (specificity + specificity − 1) were used as cut-off points in this study [23]. The calculation of IFS for each patient was based on the value of every inflammatory marker and its coefficient of the Cox analysis.

### Statistical analysis

Continuous variables were reported as mean ± standard deviation (SD), or median and interquartile range (IQR). The differences were compared by one-way analysis of variance (ANOVA) or Kruskal-Wallis H tests (K-W tests). Categorical variables were reported as frequencies and percentages, and differences in different groups were compared using the Chi-square test. OS and RFS rates were analyzed by the Kaplan-Meier (KM) curves, and the differences were compared using the log-rank test. Cox regression analyses were used to identify independent prognostic factors for OS and RFS. Survival tree analysis was applied to divide patients into three differentiated groups (high-, median-, and low-score groups) with different survival outcomes.

The AJCC 7th TNM stage, AJCC 8th TNM stage, and Child-Pugh stage were compared with the IFS system in testing the performance of prognosis prediction via the time-dependent areas under the ROC (Time-ROC) curve and akaike information criterion (AIC). Statistical analysis was performed using the SPSS® version 25.0 (IBM, Armonk, New York, USA), and R program version 3.2.0 (http://www.r-project.org/). A *P-*value < 0.05 indicated statistical significance.

## Results

### The correlation of fifteen inflammatory markers

**Fig. 1:**
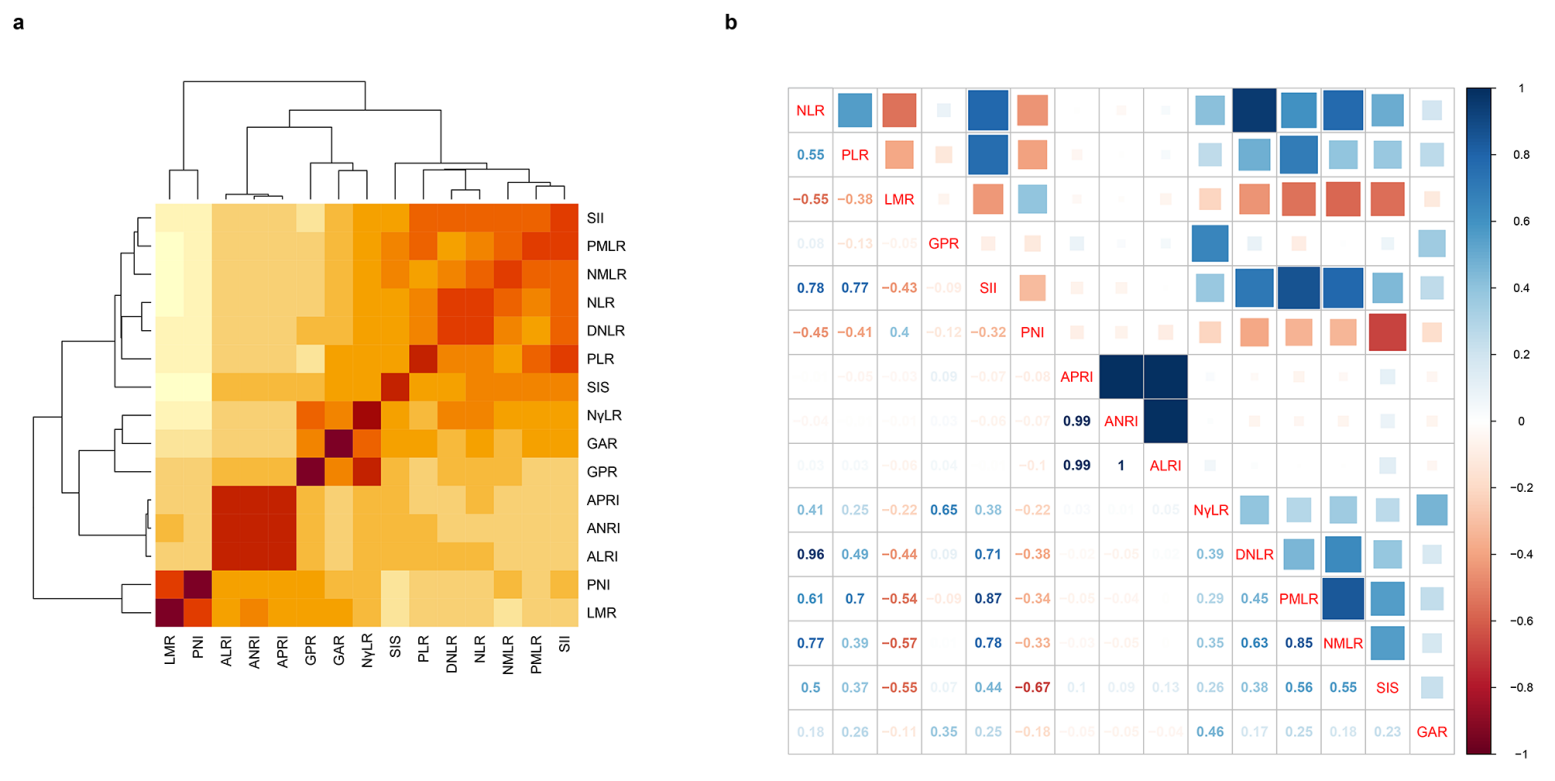
Heatmap of correlation among the fifteen inflammatory markers **(a)**. Correlation plot showing the specific correlation coefficient relationship among the fifteen inflammatory markers **(b)**

Figure 1a shows the correlation heatmap of 15 inflammatory markers in this study. The darker the yellow, the stronger the correlation (positive or negative correlation). As shown in Fig. 1b, we quantified the correlation of 15 inflammatory markers. We found that the correlation coefficient between the ANRI and ALRI was 1 point. Moreover, we also found that APRI had a strong correlation between ANRI and ALRI, with coefficients of both 0.99. In parallel, the correlations between the NLR and DNLR, SII and PMLR, and PMLR and NMLR were also strong, with coefficients of 0.96, 0.87, and 0.85, respectively.

### Construction of IFS system

**Fig. 2:**
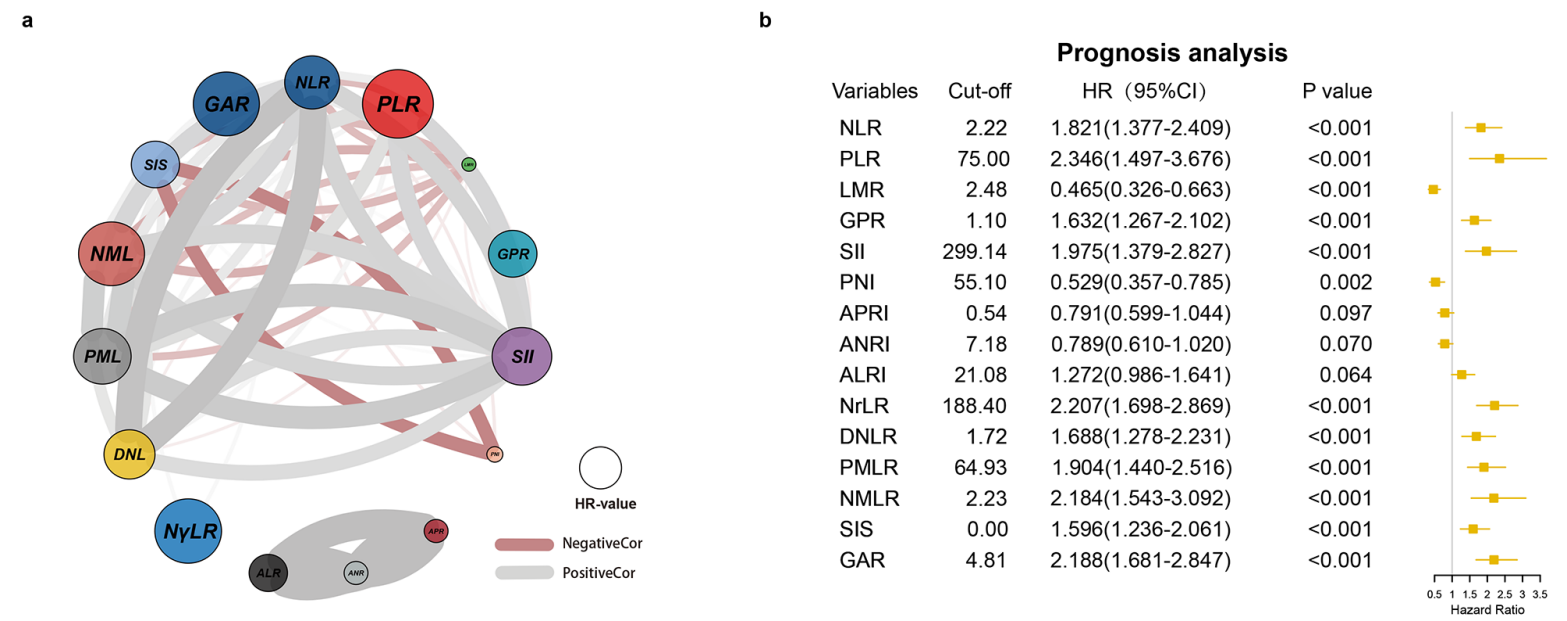
Circle correlation plot showing the relationship among the fifteen inflammatory markers **(a)**. Cut-off values of fifteen inflammatory markers and the univariable Cox prognosis analysis of those markers for OS **(b)**

Figure 2a was a circle correlation plot which illustrates the correlation among those inflammatory markers. Figure 2a also shows the influence of these markers on prognosis with their hazard ratio (HR) on OS. In Fig. 2a, link lines in two colors reflected the positive or negative correlation in 15 different inflammatory markers. The correlation coefficient values were positively correlated with the thickness of the lines. Moreover, the size of circles also reflected the HR values, which implied that the larger the circle, the greater the impact on OS.

Through the ROC curves, we found that the best cut-off values of the 15 inflammatory markers were 2.22, 75.00, 2.48, 1.10, 299.14, 55.10, 0.54, 7.18, 21.08, 188.40, 1.72, 64.93, 2.23, 0.00, and 4.81, respectively (Fig. 2b). After univariate Cox regression analysis, we found that 12 markers were significantly associated with the prognosis of patients with ICC in training cohort. A higher level of NLR (HR = 1.821, 95% confidence interval [CI]: =1.377–2.409), PLR (2.346, 1.497–3.676), GPR (1.632, 1.267–2.102), SII (1.975, 1.379–2.827), NγLR (2.207, 1.698–2.869), dNLR (1.688, 1.278–2.231), PMLR (1.904, 1.440–2.516), NMLR (2.184, 1.543–3.092), SIS (1.596, 1.236–2.061), and GAR (2.188, 1.681–2.847) were risk factors of OS, and a higher level of LMR (0.465, 0.326–0.663) and PNI (0.529, 0.357–0.785) were protective factors of OS. However, APRI, ANRI, and ALRI had no influence on OS after Cox regression analyses. Therefore, we scored every patient using the above 12 positive inflammatory markers, as illustrated in Table 1, according to the marker values and their corresponding coefficients. First, we calculated the median coefficient value of the 12 inflammatory markers for a value of 0.662. Second, we used every marker’s coefficient value to divide 0.662 and then rounded it to the whole number (> 0.5 scored 1 point, < 0.5 scored 0 point) to obtain the inflammatory coefficient. Third, a patient was scored 1 point if his or her inflammatory marker > cut-off value; otherwise, 0 points were scored. Finally, we used inflammatory-coefficient times 0 or 1 for each marker and summed the total points for every patient to form IFS.

**Table 1:** Cut-off value and IFS in 15 inflammatory markers

No.	Variables	Cut-off	HR	Coefficient	Median coefficient^†^	Inflammatory-coefficient^*^	Cut-off score^††^	IFS
1	NLR	2.22	1.821	0.6	0.662	1	0 or 1	1 or 0
2	PLR	75	2.346	0.853	0.662	1	0 or 1	1 or 0
3	LMR	2.48	0.465	-0.766	0.662	-1	0 or 1	-1 or 0
4	GPR	1.1	1.603	0.472	0.662	1	0 or 1	1 or 0
5	SII	299.14	1.975	0.68	0.662	1	0 or 1	1 or 0
6	PNI	55.1	0.529	-0.636	0.662	-1	0 or 1	-1 or 0
7	APRI	0.54	0.791	-0.235	-	-	-	-
8	ANRI	7.18	0.789	-0.238	-	-	-	-
9	ALRI	21.08	1.272	0.241	-	-	-	-
10	NrLR	188.4	2.207	0.792	0.662	1	0 or 1	1 or 0
11	dNLR	1.72	1.688	0.524	0.662	1	0 or 1	1 or 0
12	PMLR	64.93	1.904	0.644	0.662	1	0 or 1	1 or 0
13	NMLR	2.23	2.184	0.781	0.662	1	0 or 1	1 or 0
14	SIS	0	1.596	0.468	0.662	1	0 or 1	1 or 0
15	GAR	4.81	2.188	0.783	0.662	1	0 or 1	1 or 0

†† **Median coefficient**: calculated based on the 12 inflammatory markers (NLR, PLR, LMR, GPR, SII, PNI, NγLR, dNLR, PMLR, NMLR, SIS, and GAR)

*Inflammatory-coefficient: every marker’ s coefficient value is used to divide 0.662 and then be rounded to the whole number (> 0.5 scored 1 point, < 0.5 scored 0 point)

† **Cut-off score**: if the value of an inflammatory marker over its cut-off value, we score 1 point, otherwise, we score 0 point

**IFS**: inflammatory-coefficient times cut-off score

### Patients were divided into three groups according to IFS

**Fig. 3:**
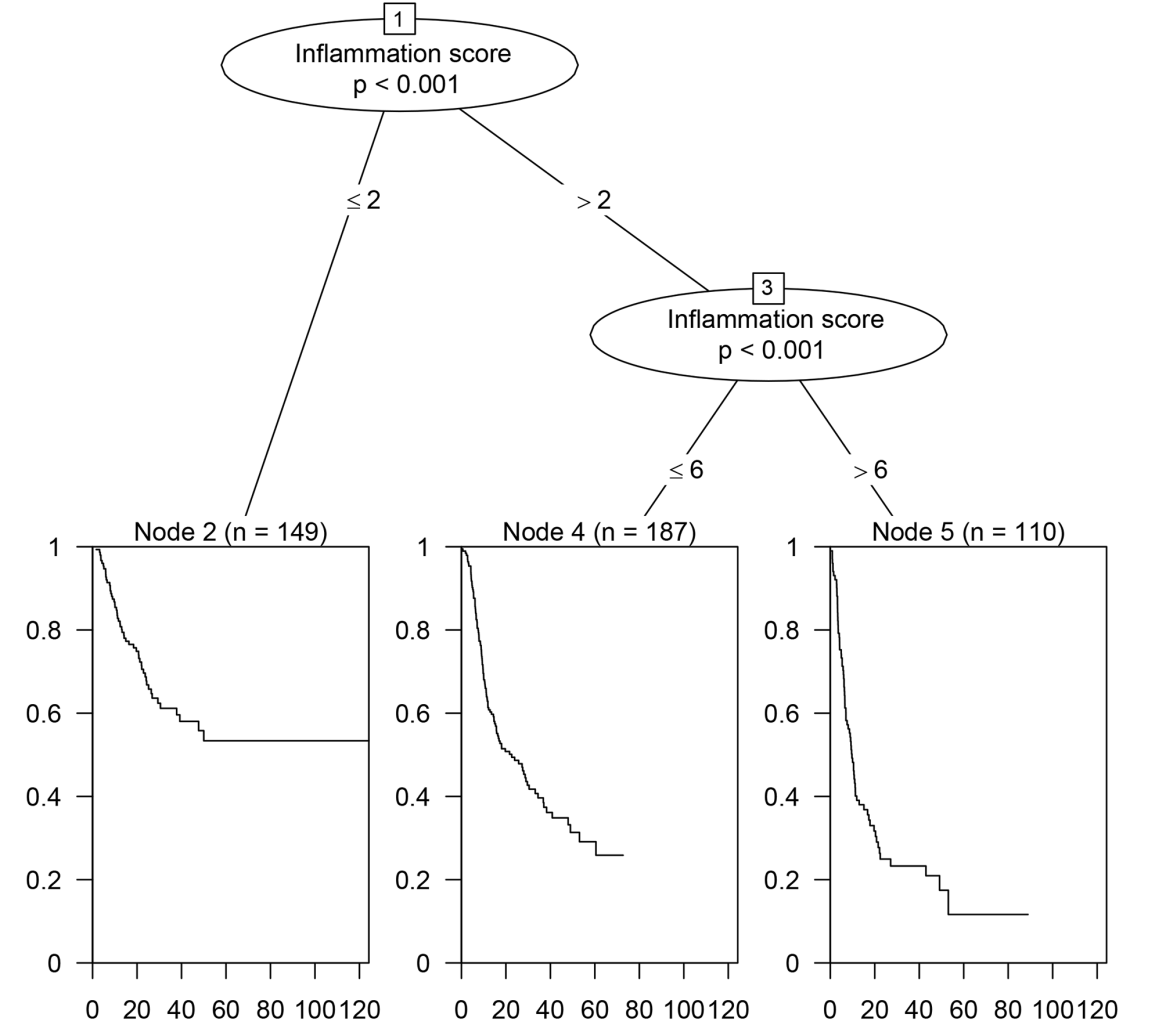
Survival tree analysis of OS in the training cohort and 2 point and 6 point were considered to be two best cut-off values

**Fig. 4:**
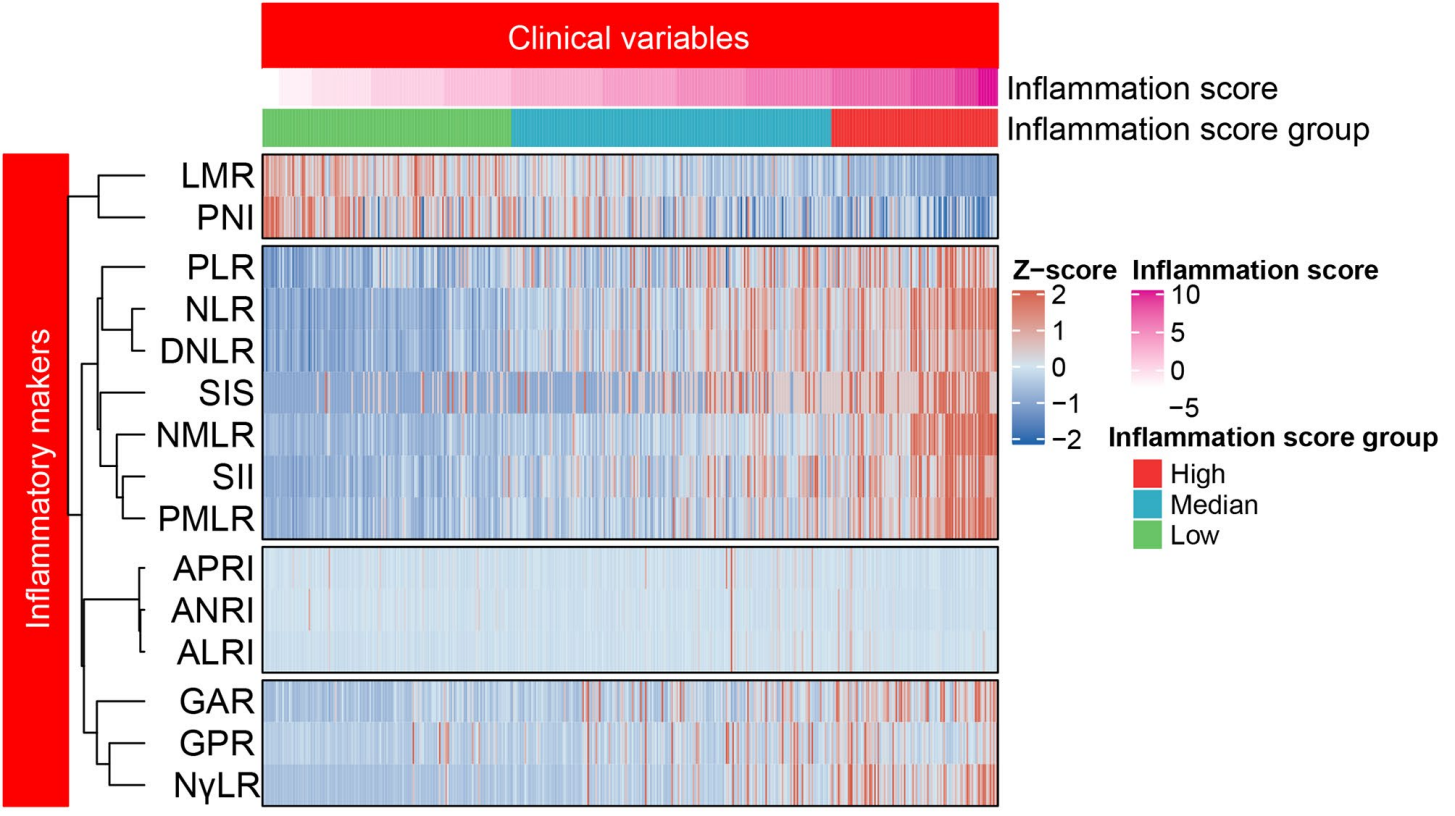
Heatmap showing the distribution of 15 inflammatory markers’ values in the training cohort according to the different IFS values and different IFS groups

As shown in Fig. 3, the OS survival tree divided the patients in training cohort into three groups with different IFS levels. The best two cut-off values were 2 and 6 points, respectively. As a result, patients with IFS ≤ 2 were assigned to the low-IFS-score group (n = 149), > 6 were assigned to the high-IFS-score group (n = 110), and the remaining patients were assigned to the median-IFS-score group (n = 187). Additionally, Fig. 4 demonstrates the heatmap showing the distribution of the values of the 15 inflammatory markers in the training cohort according to the different IFS values and different IFS groups. On the vertical axis, 15 types of inflammatory markers could be clustered into five categories.

### Baseline characteristics of the three groups in the training cohort

Table 2 shows the basic clinicopathological characteristics of three groups in the training cohort. As shown in Tables 2 and 149 patients were in the low-IFS-score group (33.4%), 187 patients were in the median-IFS-score group (41.9%), and 110 patients were in the high-IFS-score group (24.7%).

**Table 2:** Baseline clinicopathological characteristics of the three groups in the training cohort

Variables	level	Low IFS score	Median IFS score	High IFS score	P value
		N = 149	N = 187	N = 110	
Age, year (mean ± SD)		54.36 ± 10.71	53.03 ± 10.67	55.08 ± 9.18	0.220
Sex (%)	female	52 (34.9)	65 (34.8)	34 (30.9)	0.753
	male	97 (65.1)	122 (65.2)	76 (69.1)	
Hepatolithiasis (%)	no	135 (90.6)	172 (92.0)	99 (90.0)	0.826
	yes	14 (9.4)	15 (8.0)	11 (10.0)	
Diabetes (%)	no	139 (93.3)	175 (93.6)	103 (93.6)	0.992
	yes	10 (6.7)	12 (6.4)	7 (6.4)	
HBsAg (+) (%)	no	66 (44.3)	114 (61.0)	65 (59.1)	**0.006**
	yes	83 (55.7)	73 (39.0)	45 (40.9)	
Anti-HCV (%)	no	144 (96.6)	187 (100.0)	109 (99.1)	**0.027**
	yes	5 (3.4)	0 (0.0)	1 (0.9)	
Child-Pugh grade (%)	A	147 (98.7)	184 (98.4)	104 (94.5)	0.066
	B	2 (1.3)	3 (1.6)	6 (5.5)	
TBIL, mg/dL*		12.60 [9.80, 15.40]	12.00 [9.20, 16.05]	12.45 [8.95, 16.32]	0.563
ALT, IU/L*		26.00 [17.00, 39.10]	25.70 [17.30, 40.70]	28.20 [17.07, 45.43]	0.786
AST, IU/L*		25.00 [19.00, 32.00]	27.50 [20.55, 38.80]	28.10 [20.00, 41.77]	**0.041**
GGT, IU/L*		45.00 [30.00, 71.00]	74.00 [47.50, 140.50]	168.00 [97.75, 275.75]	**< 0.001**
HgB, g/L (mean ± SD)		138.45 ± 16.45	133.45 ± 17.73	128.15 ± 18.28	**< 0.001**
PT, second*		11.60 [11.10, 12.20]	11.60 [11.00, 12.10]	11.80 [11.20, 12.30]	0.125
ALB, g/L (mean ± SD)		43.69 ± 3.60	42.51 ± 3.40	40.58 ± 4.03	**< 0.001**
PLT, ×10^9^/L*		172.00 [132.00, 219.00]	195.00 [147.00, 240.50]	219.50 [175.00, 297.50]	**< 0.001**
WBC, ×10^9^/L*		5.54 [4.64, 6.97]	6.26 [5.21, 7.58]	7.38 [6.44, 9.34]	**< 0.001**
AFP, µg/L*		3.90 [2.40, 8.50]	3.50 [2.30, 8.75]	4.05 [2.42, 12.42]	0.548
CEA, µg/L*		2.30 [1.40, 3.50]	2.60 [1.50, 5.15]	3.25 [1.70, 9.38]	**0.004**
CA199, IU/mL*		25.60 [13.30, 49.40]	56.90 [14.80, 206.30]	117.10 [27.62, 1000.00]	**< 0.001**
Perioperative transfusion (%)	no	135 (90.6)	157 (84.0)	85 (77.3)	**0.013**
	yes	14 (9.4)	30 (16.0)	25 (22.7)	
Anatomic resection (%)	no	32 (21.5)	27 (14.4)	22 (20.0)	0.213
	yes	117 (78.5)	160 (85.6)	88 (80.0)	
Major resection (%)	no	124 (83.2)	131 (70.1)	71 (64.5)	**0.002**
	yes	25 (16.8)	56 (29.9)	39 (35.5)	
Resection margin ≥ 10 mm (%)	yes	21 (14.1)	20 (10.7)	6 (5.5)	0.081
	no	128 (85.9)	167 (89.3)	104 (94.5)	
Cirrhosis (%)	no	107 (71.8)	150 (80.2)	95 (86.4)	**0.015**
	yes	42 (28.2)	37 (19.8)	15 (13.6)	
Tumor diameter, cm (mean ± SD)		5.41 ± 2.47	7.04 ± 2.81	8.08 ± 2.92	**< 0.001**
Multiple tumors (%)	no	142 (95.3)	178 (95.2)	105 (95.5)	0.994
	yes	7 (4.7)	9 (4.8)	5 (4.5)	
Satellite nodules (%)	no	122 (81.9)	135 (72.2)	67 (60.9)	**0.001**
	yes	27 (18.1)	52 (27.8)	43 (39.1)	
Tumor capsule (%)	no	133 (89.3)	171 (91.4)	106 (96.4)	0.282
	complete	10 (6.7)	8 (4.3)	2 (1.8)	
	incomplete	6 (4.0)	8 (4.3)	2 (1.8)	
Tumor differentiation (%)	low	7 (4.7)	8 (4.3)	7 (6.4)	0.655
	median	140 (94.0)	178 (95.2)	103 (93.6)	
	high	2 (1.3)	1 (0.5)	0 (0.0)	
Adjacent tissue invasion (%)	no	145 (97.3)	177 (94.7)	98 (89.1)	**0.019**
	yes	4 (2.7)	10 (5.3)	12 (10.9)	
MVI (%)	no	141 (94.6)	163 (87.2)	94 (85.5)	**0.030**
	yes	8 (5.4)	24 (12.8)	16 (14.5)	
LNM (%)	no	140 (94.0)	158 (84.5)	78 (70.9)	**< 0.001**
	yes	9 (6.0)	29 (15.5)	32 (29.1)	

**Abbreviations**: HBsAg, hepatitis B surface antigen; HCV, hepatitis C virus; TBIL, total bilirubin; ALB, albumin; ALT, alanine transaminase; AST, aspartate aminotransferase; GGT, gamma-glutamyl transpeptidase; PT, prothrombin time; PLT, platelet; AFP, alpha-fetoprotein; CEA, carcinoembryonic antigen; CA19-9, carbonhydrateantigen19-9; MVI, microvascular invasion; LNM, lymph node metastasis

* Quantitative variables shown with median and interquartile range (IQR)

When we compared the differences in characteristics, we found that major resection (*P* = 0.002), perioperative transfusion (*P* = 0.013), HBsAg (+) (*P* = 0.006), anti-HCV (*P* = 0.027), satellite nodules (*P* = 0.001), cirrhosis (*P* = 0.015), lymph node metastasis (LNM) (*P* < 0.001), MVI (*P* = 0.030), adjacent tissue invasion (*P* = 0.019), AST (*P* = 0.041), GGT (*P* < 0.001), HgB (*P* < 0.001), ALB (*P* < 0.001), PLT (*P* < 0.001), WBC (*P* < 0.001), CEA (*P* = 0.004), CA199 (*P* < 0.001), and tumor diameter (*P* < 0.001) were significantly different among the three groups.

### Survival analysis

**Fig. 5:**
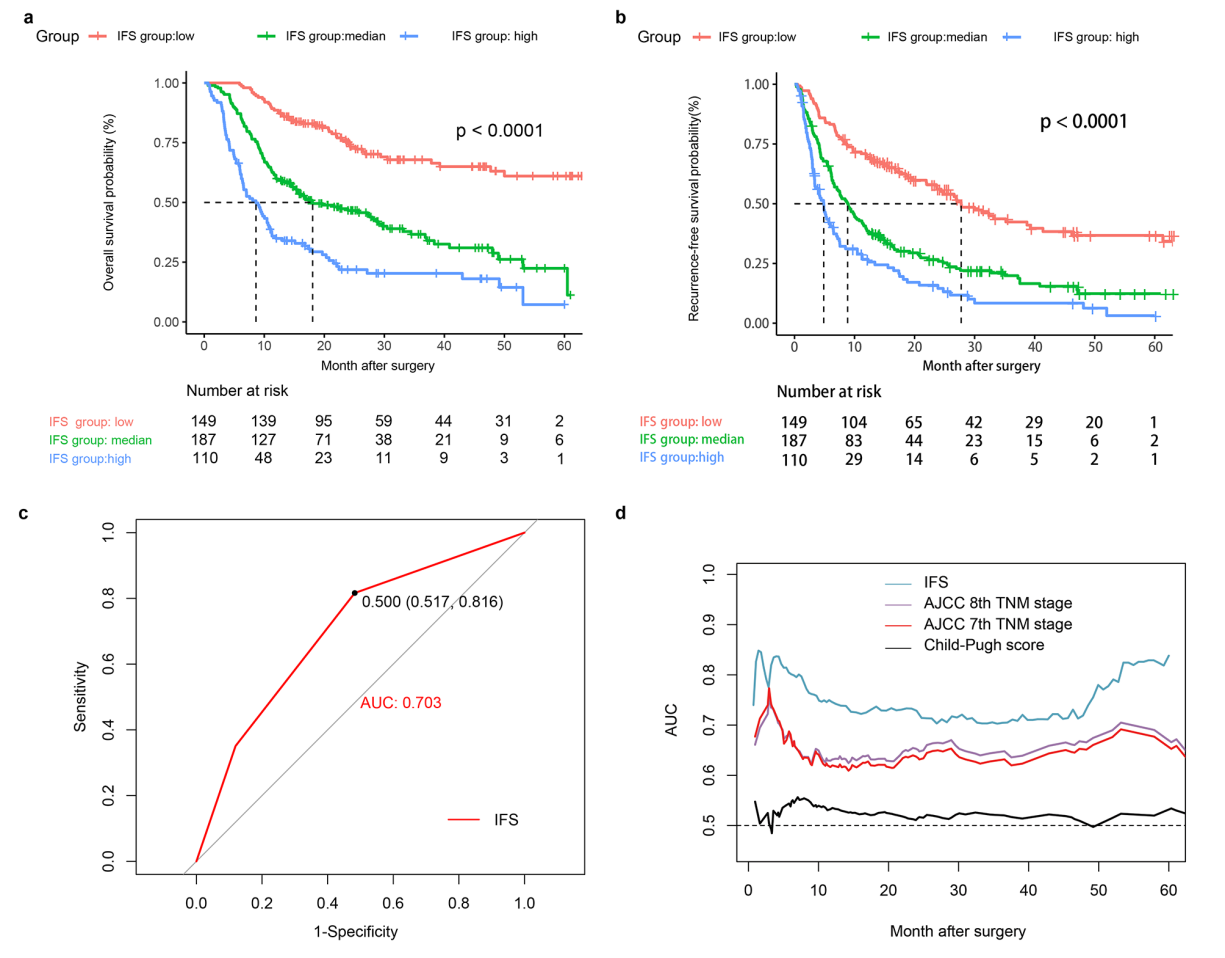
KM curves of OS and RFS for patients with ICC in the training cohort (**a and b**). ROC curves of IFS system (**c**), and the time-dependent ROC curves for IFS system and other clinical staging systems in the training cohorts (**d**)

The KM curves in Fig. 5a and b showed that significantly different OS and RFS rates were observed among the three groups (both *P* < 0.0001). The postoperative 1-, 3-, and 5-year OS rates of the low-IFS group (88.6%, 67.7%, and 60.7%, respectively) were significantly higher than those of the median-IFS group (59.9%, 37.1%, and 21.5%, respectively), and the high-IFS group (34.9%, 20.9%, and 9.2%, respectively). Similarly, the postoperative 1-, 3-, and 5-year RFS rates of the low-IFS group (69.9%, 42.9%, and 36.9%, respectively) were significantly higher than those of the median-IFS group (39.8%, 20.2%, and 12.4%, respectively), and the high-IFS group (27.6%, 9.2%, and 3.9%, respectively).

### Independent prognostic factors for OS and RFS

The variables listed in Table 2 were included into the Cox regression analysis. All significant covariates (P < 0.050) in the univariate Cox analysis were included in the multivariable Cox analysis. Table 3 shows the results of univariate and multivariate analyses results for OS. We found that anatomic resection (HR = 1.578, 95%CI = 1.085–2.295, P = 0.017), high level of CA19-9 (HR = 1.000, 95%CI = 1.0001–1.001, P = 0.010), satellite nodules (HR = 1.448, 95%CI = 1.095–1.915, P = 0.009), LNM (HR = 1.422, 95%CI = 1.038–1.949, P = 0.028), MVI (HR = 1.014, 95%CI = 1.101–2.291, P = 0.014), and high-IFS group (HR = 2.106, 95%CI = 1.682–2.637, P < 0.001) were independent risk factors for OS.

**Table 3:** Univariable and multivariable analyses for OS in the training cohort

	Univariable			Multivariable		
Variables	HR	95%CI	P value	HR	95%CI	P value
Age, year	1.005	0.993–1.017	0.453			
Sex, male vs. female	1.055	0.808–1.377	0.694			
Hepatolithiasis, yes vs. no	1.266	0.824–1.945	0.282			
Diabetes, yes vs. no	0.921	0.555–1.530	0.751			
HBsAg (+), yes vs. no	0.816	0.633–1.052	0.116			
Anti-HCV, yes vs. no	0.749	0.239–2.343	0.619			
Child-Pugh grade, B vs. A	0.446	0.143–1.394	0.165			
TBIL, mg/dL	0.999	0.993–1.005	0.761			
ALT, IU/L	1.001	0.999–1.002	0.081			
AST, IU/L	1.001	1.000-1.002	0.042			
GGT, IU/L	1.001	1.000-1.002	0.011			
HgB, g/L	0.991	0.984–0.998	0.009			
PT, second	1.058	0.956–1.172	0.274			
ALB, g/L	0.950	0.92–0.981	0.002			
PLT, ×10^9^/L	1.002	1.000-1.003	0.034			
WBC, ×10^9^/L	1.075	1.014–1.139	0.015			
AFP, µg/L	1.000	0.999–1.001	0.615			
CEA, µg/L	1.000	0.999–1.001	0.142			
CA19-9, IU/mL	1.001	1.000-1.002	< 0.001	1.000	1.0001–1.001	0.010
Perioperative transfusion, yes vs. no	1.354	0.974–1.883	0.071			
Anatomic resection, yes vs. no	1.704	1.175–2.472	0.005	1.578	1.085–2.295	0.017
Major resection, yes vs. no	1.553	1.186–2.033	0.001			
Resection margin < 10 mm, yes vs. no	1.833	1.134–2.963	0.012			
Cirrhosis, yes vs. no	0.854	0.623–1.171	0.327			
Tumor diameter, cm	1.117	1.074–1.162	< 0.001			
Multiple tumors, yes vs. no	0.816	0.433–1.538	0.530			
Satellite nodules, yes vs. no	1.997	1.537–2.595	< 0.001	1.448	1.095–1.915	0.009
Tumor capsule, yes vs. no	0.620	0.421–0.914	0.016			
Tumor differentiation, high vs. median vs. low	1.410	0.809–2.458	0.225			
Adjacent tissue invasion, yes vs. no	1.570	0.944–2.611	0.082			
MVI, yes vs. no	1.794	1.254–2.566	0.001	1.587	1.101–2.291	0.014
LNM, yes vs. no	2.179	1.618–2.935	< 0.001	1.422	1.038–1.949	0.028
IFS, high vs. median vs. low	2.320	1.955–2.753	< 0.001	2.106	1.682–2.637	< 0.001

**Abbreviations**: HBsAg, hepatitis B surface antigen; HCV, hepatitis C virus; TBIL, total bilirubin; ALB, albumin; ALT, alanine transaminase; AST, aspartate aminotransferase; GGT, gamma-glutamyl transpeptidase; PT, prothrombin time; PLT, platelet; AFP, alpha-fetoprotein; CEA, carcinoembryonic antigen; CA19-9, carbonhydrateantigen19-9; MVI, microvascular invasion; LNM, lymph node metastasis; IFS, inflammation score

Additionally, Table 4 shows the results of univariate and multivariate Cox analyses for RFS. Multivariate analyses identified high level of CA19-9 (HR = 1.001, 95%CI = 1.000-1.008, *P* = 0.011), larger tumor diameter (HR = 1.059, 95%CI = 1.015–1.105, *P* = 0.008), satellite nodules (HR = 1.419, 95%CI = 1.098–1.834, *P* = 0.007), LNM (HR = 1.406, 95%CI = 1.040–1.901, *P* = 0.027), MVI (HR = 1.664, 95%CI = 1.182–2.342, *P* = 0.003), and high-IFS group (HR = 1.572, 95%CI = 1.322–1.87, *P* < 0.001) were independent risk factors for RFS.

**Table 4:** Univariable and multivariable analyses for RFS in the training cohort

	Univariable			Multivariable		
Variables	HR	95%CI	P value	HR	95%CI	P value
Age, year	0.997	0.986–1.008	0.586			
Sex, male vs. female	0.938	0.745–1.180	0.583			
Hepatolithiasis, yes vs. no	0.995	0.675–1.467	0.980			
Diabetes, yes vs. no	0.770	0.479–1.240	0.283			
HBsAg (+), yes vs. no	0.859	0.688–1.071	0.177			
Anti-HCV, yes vs. no	0.313	0.078–1.257	0.101			
Child-Pugh grade, B vs. A	0.754	0.374–1.523	0.432			
TBIL, mg/dL	1.001	0.997–1.004	0.655			
ALT, IU/L	0.999	0.996–1.001	0.335			
AST, IU/L	1.000	0.998–1.001	0.656			
GGT, IU/L	1.000	0.999–1.001	0.070			
HgB, g/L	0.994	0.988-1.000	0.049			
PT, second	1.036	0.940–1.141	0.479			
ALB, g/L	0.986	0.959–1.013	0.311			
PLT, ×10^9^/L	1.002	1.000-1.003	0.037			
WBC, ×10^9^/L	1.064	1.010–1.122	0.019			
AFP, µg/L	1.001	0.999–1.002	0.151			
CEA, µg/L	1.000	0.999–1.001	0.110			
CA19-9, IU/mL	1.001	1.0006–1.0012	< 0.001	1.001	1.000-1.008	0.011
Perioperative transfusion, yes vs. no	1.152	0.857–1.548	0.348			
Anatomic resection, yes vs. no	1.289	0.966–1.720	0.085			
Major resection, yes vs. no	1.299	1.017–1.658	0.036			
Resection margin < 10 mm, yes vs. no	1.439	0.987–2.096	0.057			
Cirrhosis, yes vs. no	0.788	0.596–1.043	0.096			
Tumor diameter, cm	1.117	1.079–1.158	< 0.001	1.059	1.015–1.105	0.008
Multiple tumors, yes vs. no	1.039	0.637–1.694	0.879			
Satellite nodules, yes vs. no	1.970	1.557–2.494	< 0.001	1.419	1.098–1.834	0.007
Tumor capsule, yes vs. no	0.750	0.568–0.989	0.042			
Tumor differentiation, high vs. median vs. low	1.019	0.662–1.569	0.93			
Adjacent tissue invasion, yes vs. no	1.810	1.160–2.825	0.009			
MVI, yes vs. no	1.955	1.400–2.730	< 0.001	1.664	1.182–2.342	0.003
LNM, yes vs. no	1.998	1.511–2.643	< 0.001	1.406	1.040–1.901	0.027
IFS, high vs. median vs. low	1.857	1.603–2.153	< 0.001	1.572	1.322–1.87	< 0.001

**Abbreviations**: HBsAg, hepatitis B surface antigen; HCV, hepatitis C virus; TBIL, total bilirubin; ALB, albumin; ALT, alanine transaminase; AST, aspartate aminotransferase; GGT, gamma-glutamyl transpeptidase; PT, prothrombin time; PLT, platelet; AFP, alpha-fetoprotein; CEA, carcinoembryonic antigen; CA19-9, carbonhydrateantigen19-9; MVI, microvascular invasion; LNM, lymph node metastasis; IFS, inflammation score

### Comparison of different scoring systems

The ROC curve of IFS demonstrated in Fig. 5c had an AUC value of 0.703 (95%CI = 0.658–0.748), which suggested that the IFS system had a good prognosis predictive performance for OS in the training cohort.

Time-ROC curves were established to compare the performance of the IFS system with the other three scoring systems (Fig. 5d), which showed that the AUC value of the IFS from 0 to 60 months was better than that of the other three scoring systems in the training cohort. The AIC value of the IFS was also obviously lower than others in the training cohort. The comparison results are listed in Table 5, and we found that the survival predictive ability of the IFS was significantly better than that of the Child-Pugh stage (*P* < 0.0001), AJCC 7th TNM stage (*P* < 0.0001), and AJCC 8th TNM stage (*P* < 0.0001).

**Table 5:** Comparison of the IFS with other clinical staging systems in the training cohort

Clinical staging systems	C-index (95%CI)	Change (95%CI)	P value	AIC value
IFS	0.684 (0.654–0.714)			2646.467
AJCC 7th TNM Stage	0.607 (0.574–0.641)	-0.077 (-0.111 — -0.035)	**< 0.0001**	2701.532
AJCC 8th TNM Stage	0.614 (0.581–0.648)	-0.070 (-0.106 — -0.027)	**0.0005**	2697.023
Child-Pugh Stage	0.505 (0.494–0.516)	-0.179 (-0.212 — -0.144)	**< 0.0001**	2740.608

**Abbreviation**: AJCC: American Joint Committee on Cancer; AIC: Akaike information criterion

### The validation of IFS system

**Fig. 6:**
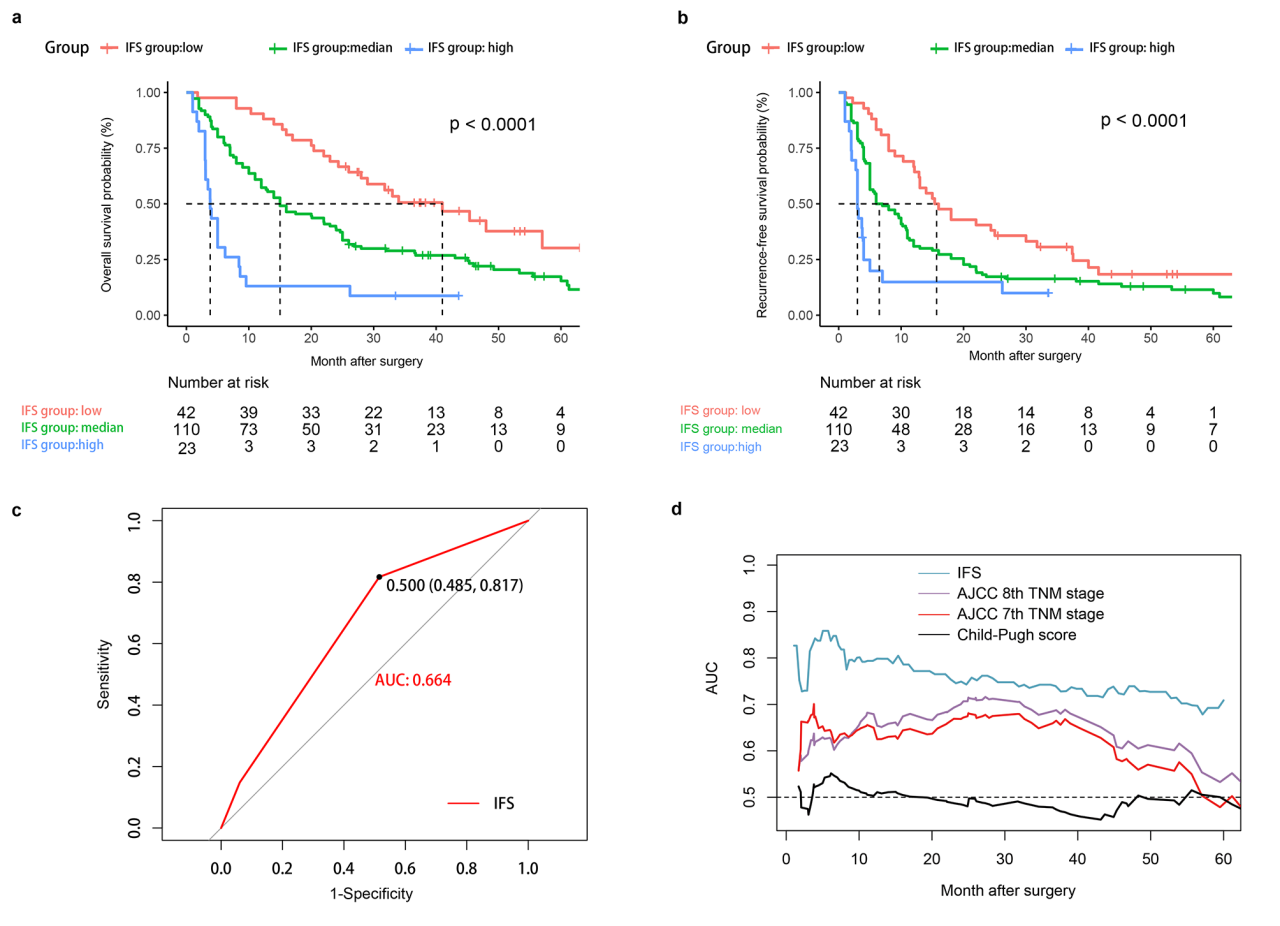
KM curves of OS and RFS for patients with ICC in the internal validation cohort (**a and b**). ROC curves of IFS system (**c**), and the time-dependent ROC curves for IFS system and other clinical staging systems in the internal validation cohort (**d**)

The internal validation cohort consisted of 175 patients, and the basic clinicopathologic characteristics were listed in Table 6. KM curves illustrated that patients in the high-IFS group presented with poorer OS and RFS rates compared with those in the median-IFS and low-IFS groups. The 1-, 3-, and 5-year OS rates were 13.0%, 7.8%, and 0.0% for the high-IFS group, 60.9%, 28.6%, and 16.8% for the median-IFS group, and 90.5%, 51.1%, and 32.3% for the low-IFS group, respectively (*P* < 0.0001). Moreover, the 1-, 3-, and 5-year RFS rates were 15.5%, 7.8%, and 0.0% for the high-IFS group, 33.6%, 16.3%, and 11.4% for the median-IFS group, and 69.0%, 30.3%, and 17.6% for the low-IFS group (*P* < 0.0001) (Fig. 6a and b).

**Table 6:** Baseline clinicopathological characteristics of the three groups in the internal validation cohort

Variables	level	Low IFS score	Median IFS score	High IFS score	P value
		N = 42	N = 110	N = 23	
Age, year (mean ± SD)		58.12 ± 9.78	56.56 ± 10.68	58.87 ± 10.80	0.521
Sex (%)	female	14 (33.3)	34 (30.9)	6 (26.1)	0.833
	male	28 (66.7)	76 (69.1)	17 (73.9)	
Hepatolithiasis (%)	no	42 (100.0)	106 (96.4)	22 (95.7)	0.436
	yes	0 (0.0)	4 (3.6)	1 (4.3)	
Diabetes (%)	no	38 (90.5)	101 (91.8)	22 (95.7)	0.758
	yes	4 (9.5)	9 (8.2)	1 (4.3)	
HBsAg (+) (%)	no	22 (52.4)	67 (60.9)	15 (65.2)	0.526
	yes	20 (47.6)	43 (39.1)	8 (34.8)	
Anti-HCV (%)	no	42 (100.0)	110 (100.0)	22 (95.7)	**0.036**
	yes	0 (0.0)	0 (0.0)	1 (4.3)	
Child-Pugh grade (%)	A	37 (88.1)	104 (94.5)	17 (73.9)	**0.027**
	B	2 (4.8)	4 (3.6)	4 (17.4)	
TBIL, mg/dL*		12.45 [10.03, 16.17]	11.90 [9.83, 14.35]	10.30 [8.85, 12.25]	0.153
ALT, IU/L*		18.50 [14.00, 25.90]	21.90 [14.00, 31.08]	28.00 [15.75, 46.00]	0.151
AST, IU/L*		22.15 [17.00, 27.88]	22.95 [17.25, 31.75]	31.80 [23.05, 42.00]	**0.012**
GGT, IU/L*		49.00 [28.25, 86.25]	73.50 [38.25, 154.75]	92.00 [55.00, 199.00]	0.079
HgB, g/L (mean ± SD)		136.05 ± 15.16	134.75 ± 13.86	124.65 ± 18.64	**0.007**
PT, second*		11.55 (1.17)	11.65 (0.90)	12.22 (1.19)	**0.027**
ALB, g/L (mean ± SD)		42.69 ± 5.27	42.36 ± 3.37	40.00 ± 3.78	**0.021**
PLT, ×10^9^/L*		162.00 [133.50, 199.00]	186.50 [146.25, 242.50]	185.00 [136.50, 223.00]	0.054
WBC, ×10^9^/L*		5.98 [4.66, 6.82]	6.08 [5.03, 7.46]	6.13 [5.04, 8.27]	0.428
AFP, µg/L*		3.50 [1.90, 6.05]	3.05 [2.12, 5.70]	6.60 [2.40, 21.00]	0.208
CEA, µg/L*		2.30 [1.40, 3.65]	2.90 [2.00, 6.10]	3.30 [1.55, 4.70]	**0.024**
CA199, IU/mL*		36.45 [18.35, 124.85]	69.75 [24.20, 670.12]	45.20 [16.65, 171.60]	0.104
Perioperative transfusion (%)	no	37 (88.1)	96 (87.3)	19 (82.6)	0.804
	yes	5 (11.9)	14 (12.7)	4 (17.4)	
Anatomic resection (%)	no	30 (71.4)	71 (64.5)	13 (56.5)	0.472
	yes	12 (28.6)	39 (35.5)	10 (43.5)	
Major resection (%)	no	30 (71.4)	74 (67.3)	18 (78.3)	0.559
	yes	12 (28.6)	36 (32.7)	5 (21.7)	
Cirrhosis (%)	no	31 (73.8)	89 (80.9)	15 (65.2)	0.223
	yes	11 (26.2)	21 (19.1)	8 (34.8)	
Tumor diameter, cm (mean ± SD)		5.55 ± 3.54	6.58 ± 3.60	8.02 ± 3.76	**0.031**
Multiple tumors (%)	no	34 (81.0)	70 (63.6)	18 (78.3)	0.073
	yes	8 (19.0)	40 (36.4)	5 (21.7)	
Satellite nodules (%)	no	27 (64.3)	58 (52.7)	16 (69.6)	0.203
	yes	15 (35.7)	52 (47.3)	7 (30.4)	
Tumor capsule (%)	no	40 (95.2)	100 (90.9)	21 (91.3)	0.747
	complete	1 (2.4)	7 (6.4)	2 (8.7)	
	incomplete	1 (2.4)	3 (2.7)	0 (0.0)	
Tumor differentiation (%)	low	3 (7.1)	9 (8.2)	3 (13.0)	0.466
	median	37 (88.1)	96 (87.3)	17 (73.9)	
	high	2 (4.8)	5 (4.5)	3 (13.0)	
Adjacent tissue invasion (%)	no	40 (95.2)	105 (95.5)	20 (87.0)	0.267
	yes	2 (4.8)	5 (4.5)	3 (13.0)	
MVI (%)	no	35 (83.3)	88 (80.0)	21 (91.3)	0.425
	yes	7 (16.7)	22 (20.0)	2 (8.7)	
LNM (%)	no	40 (95.2)	96 (87.3)	19 (82.6)	0.242
	yes	2 (4.8)	14 (12.7)	4 (17.4)	

**Abbreviations**: HBsAg, hepatitis B surface antigen; HCV, hepatitis C virus; TBIL, total bilirubin; ALB, albumin; ALT, alanine transaminase; AST, aspartate aminotransferase; GGT, gamma-glutamyl transpeptidase; PT, prothrombin time; PLT, platelet; AFP, alpha-fetoprotein; CEA, carcinoembryonic antigen; CA19-9, carbonhydrateantigen19-9; MVI, microvascular invasion; LNM, lymph node metastasis

* Quantitative variables shown with median and interquartile range (IQR)

The ROC curve of IFS in the internal validation cohort was shown in Fig. 6c, and the AUC value was 0.664 (95%CI = 0.569–0.760). Moreover, we also compared the prognosis predictive performance of several scoring systems through time–ROC curves, which showed that the survival predictive ability of IFS was still better than that of the Child-Pugh stage, AJCC 7th TNM stage, and AJCC 8th TNM stage (Fig. 6d).

## Discussion

In this study, we developed a new inflammatory score system, abbreviated as the IFS system based on the weight of different inflammatory markers impact on OS in the training cohort, to make a prognostic prediction for ICC after surgery. When patients were divided into high-, median-, and low-IFS groups according to different IFS levels, we found that patients in the high-IFS group had worse OS and RFS rates than those in the median- and low-IFS groups, and the same outcomes were observed in the internal validation cohort. In addition, we found that a high IFS score was an independent risk factor for OS and RFS after Cox regression analyses in the training cohort. More importantly, the IFS score had a good performance of prognostic prediction in ICC with an AUC value of 0.703 and 0.664 in the training and internal validation cohorts, respectively, which were better than those of other scoring systems. In summary, our newly constructed IFS system is a good prognostic predictor for ICC, and a high IFS is significantly associated with worse OS and RFS in patients after hepatectomy.

Extensive studies have revealed that inflammatory responses play decisive roles in tumor development and progression [24]. Over time, an increasing number of inflammation-related indicators have been constructed to serve as predictors of survival after therapies in many tumors. SIS, PNI, NLR, PLR, and Glasgow prognostic score (GPS) are all markers that have been proven to have good prognostic predictive abilities in malignant liver disease [25, 26]. However, most studies used only one or two inflammatory markers in their studies to predict survival in patients with ICC. Inconsistent with these findings, we are the first to use 12 common inflammatory markers as an integrated indicator (known as IFS) to predict survival for patients with ICC. Additionally, the establishment of IFS is based on the weight of impact of every inflammatory marker on OS so that we could guarantee that the scoring system is more reasonable and comprehensive.

In our study, we included 15 inflammatory markers and finally selected 12 markers, including NLR, PLR, LMR, GPR, SII, PNI, NγLR, dNLR, PMLR, NMLR, SIS, and GAR, that were associated with survival. We then used them to build an IFS system. Those markers are a series of scoring systems that can reflect the inflammation-related characteristics of ICC. Previous studies have found that about 25% of cancers are associated with chronic inflammation, which is caused by infections or inflammatory conditions, such as hepatitis infection or prostatitis. Moreover, the existence of inflammatory cells or mediators not only participates in promotion of angiogenesis, extracellular matrix restructuring, and pre-metastatic niche formation, but also can induce some non-epidemiological characteristics in tumors [27]. Detailed exploration of the forms of cancer-related inflammation is a useful approach to discover important prognostic markers for malignant tumors in daily clinical practice. Regulating the systemic inflammatory response might become an important therapeutic target for the tumor itself in future.

Recently, some studies have demonstrated the prognostic utility of NLR, PLR, and LMR in patients with ICC after surgery [26, 28, 29]. In our study, we found that elevated NLR and PLR were negative predictors of OS in the training cohort, while low LMR was a protective factor of OS, which was consistent with the aforementioned studies. In addition, PNI and γ‑glutamyl transferase-associated enzymes, such as GPR and GAR, have been reported to be associated with the prognosis of ICC [30–32]. Our study also revealed that GPA, GAR, and PNI were parameters that were significantly associated with the prognosis of ICC in the training cohort. As we know, a high level of GGT usually reflects the disease severity of bile tract disarrangement, and low PNI levels can reflect a patient’s nutritional status in ICC.

In 2019, Wang et al. reported that in early recurrent HCC, only the SII (P < 0.001) was an independent predictor for post-recurrence survival in multivariate analyses [33]. Another study also found that increased SII was associated with decreased survival (P < 0.01) in ICC [8]. The SII score is based on lymphocytes, neutrophils, and platelets. Known studies have demonstrated that neutrophils and platelets can promote tumor cell proliferation and metastasis [34, 35]. Lymphocytes can lead to cell apoptosis and suppress cancer cell proliferation, migration, and invasion [36]. All the above studies supported our findings that SII was a risk factor for ICC survival after surgery.

Li et al. found that in 724 patients with HCC undergoing curative resection, increased NγLR is an independent risk factor for OS and progression-free survival [37]. The univariate Cox analysis result in our study illustrated that NγLR could significantly predict worse survival in patients with ICC, so, it was included into the IFS system. Additionally, the dNLR is composed of only the WBC and neutrophil counts. Previous studies have found that elevated dNLR is a negative factor of survival in HCC, pancreatic cancer, and colorectal cancer [38–41]. However, several studies also concluded that dNLR was not an independent risk factor for survival in renal cell carcinoma [42] and gastric cancer [43]. In our study, we found that a high dNLR level, which was a part of the IFS system, was a risk factor for OS in ICC.

In 2018, Liao et al. found that NMLR and PMLR were risk factors for OS and RFS in patients with HCC [44], which was consistent with ours in patients with ICC. More importantly, a previous study from China also found that NMLR and PMLR were associated with OS and RFS in gastric cancer [45]. NMLR and PMLR are inflammatory markers involved in various immune cells, which might reflect the synergistic effects or complex interaction between different immune cells in the tumor milieu. In 2019, another study from China found that the SIS, which is based on preoperative serum CA19-9 and LMR, was a powerful prognostic biomarker in ICC. In their study, Zhang et al. found that SIS was an independent risk factor for OS and TTR. Moreover, high SIS was significantly associated with aggressive tumor behavior, such as high TNM stage, large tumor size, multiple tumors, and LNM [20]. In our study, we found that SIS was a risk factor for OS in the training cohort, and IFS was an independent risk factor for OS and RFS in ICC.

Our study had several limitations. First, inflammatory marker such as C-reactive protein (CRP) was not included in this analysis because preoperative CRP is not a routine test in our institution, so we lack the data to complete the relevant statistical analysis. CRP is considered to have a higher sensitivity, but a poorer specificity, and CRP values are susceptible to many factors. Therefore, we believe that CRP is not suitable for being an excellent inflammatory marker parameter to predict the prognosis of patients with ICC. Second, collinearity exists on some inflammatory markers, which is a complex problem to avoid, especially including too many variates. In this study, we combined all the inflammatory markers for a single indicator (IFS) according to their prognostic weights to assess the role of inflammatory markers systematically and comprehensively. In this manner, the effect of covariance can be reduced to a minimum level. Third, we only used tumor differentiation instead of the histological type (mass-forming, periductal-infiltrating, intraductal-growing), which is a shortcoming of this study. Finally, it was a retrospective study. Further validation from more external cohorts is needed.

## Conclusion

Collectively, the IFS established based on 12 common inflammatory markers in our study is a potentially powerful prognostic predictor in patients with ICC after curative resection. IFS is an independent risk factor for OS and RFS in ICC and has a more accurate predictive ability than the AJCC 7th edition, AJCC 8th edition, and Child-Pugh score, which underlines the necessity of considering IFS as an important prognostic factor for ICC in daily clinical practice.

## Data Availability

The datasets used and/or analysed during the current study are available from the corresponding author on reasonable request.

## Abbreviations

IFS: Inflammation score
ICC: Intrahepatic cholangiocarcinoma
KM: Kaplan-Meier
OS: Overall survival
RFS: Recurrence-free survival
AUC: Area under the curve
AIC: Akaike information criterion
AJCC: American Joint Committee on Cancer
HCC: Hepatocellular carcinoma
SIS: Systemic inflammation score
NLR: Neutrophil-to-lymphocyte ratio
LMR: Lymphocyte to monocyte ratio
ECOG: Eastern Cooperative Oncology Group
CA19-9: Carbohydrate antigen 19–9
CEA: Carcinoembryonic antigen
AFP: Alpha-fetoprotein
CT: Computerized tomography
MRI: Magnetic resonance imaging
MVI: Microvascular invasion
NLR: Neutrophil-to-lymphocyte ratio
PLR: Platelet-to-lymphocyte ratio
LMR: Lymphocyte-to-monocyte
GPR: Gamma‑glutamyl transpeptidase-to-platelet ratio
SII: Systemic immune-inflammation index
PNI: Prognostic nutritional index
APRI: Aspartate aminotransferase-to-platelet ratio index
ANRI: Aspartate aminotransferase-to-neutrophil ratio index
ALRI: Aspartate aminotransferase-to-lymphocyte ratio index
NγLR: Neutrophil times γ-GT]/lymphocyte
dNLR: Derived neutrophil-to-lymphocyte ratio
PMLR: Platelet‑monocyte‑lymphocyte ratio
NMLR: Neutrophil‑monocyte‑lymphocyte ratio
SIS: Inflammation score
GAR: Globulin-to-albumin ratio
CRP: C-reactive protein
LNM: lymph node metastasis
ROC: Receiver operating characteristic
